# Acute Abdominal Pain in Pregnancy: A Rare Life-Threatening Condition

**DOI:** 10.7759/cureus.104560

**Published:** 2026-03-02

**Authors:** Ahmed Kassem, Yaser Aljaro, Waleed Salem, Salah Almughalles

**Affiliations:** 1 Emergency Department, Hamad Medical Corporation, Doha, QAT; 2 Emergency Medicine Department, Hamad Medical Corporation, Doha, QAT; 3 Medical Imaging Department, Hamad Medical Corporation, Doha, QAT

**Keywords:** abdominal ct, abdominal pain, acute abdomen, aneurysm, angiomyolipoma, digital subtraction angiography(dsa), fetal-maternal mortality, pregnancy, pseudoaneurysm, renal colic mimic

## Abstract

Acute abdominal pain during pregnancy has a broad differential diagnosis, and the diagnostic workup in the emergency department can be challenging, often requiring coordination across multiple specialties. Multidisciplinary collaboration involving surgery, gynecology/obstetrics, and maternal-fetal medicine is essential in selected cases to ensure timely diagnosis and appropriate management. We present the case of a pregnant woman with acute left flank abdominal pain, notable for its diagnostic complexity in the emergency department, the interdisciplinary decision‑making required, its subsequent management, its final diagnosis, and the potential underlying etiology.

## Introduction

Renal angiomyolipoma (AML) is an uncommon renal tumor, accounting for a small proportion of solid renal masses encountered in clinical practice. It is a benign mesenchymal neoplasm composed histologically of a variable admixture of mature adipose tissue, dysmorphic blood vessels, and smooth muscle fibers. The incidence of AML is approximately 0.3% in the general population and is even lower during pregnancy [1]. Most AMLs follow an indolent course and are discovered incidentally during cross-sectional imaging performed for unrelated reasons. They occur more frequently in women than in men and are typically diagnosed in middle age [2].

AMLs are most commonly located in the kidney, although other sites, including the liver, spleen, uterus, and fallopian tubes, have been reported. During pregnancy, these tumors appear to have an increased growth rate, which raises the risk of rupture and significant hemorrhage, potentially resulting in both maternal and fetal morbidity and mortality.

## Case presentation

**Figure 1:**
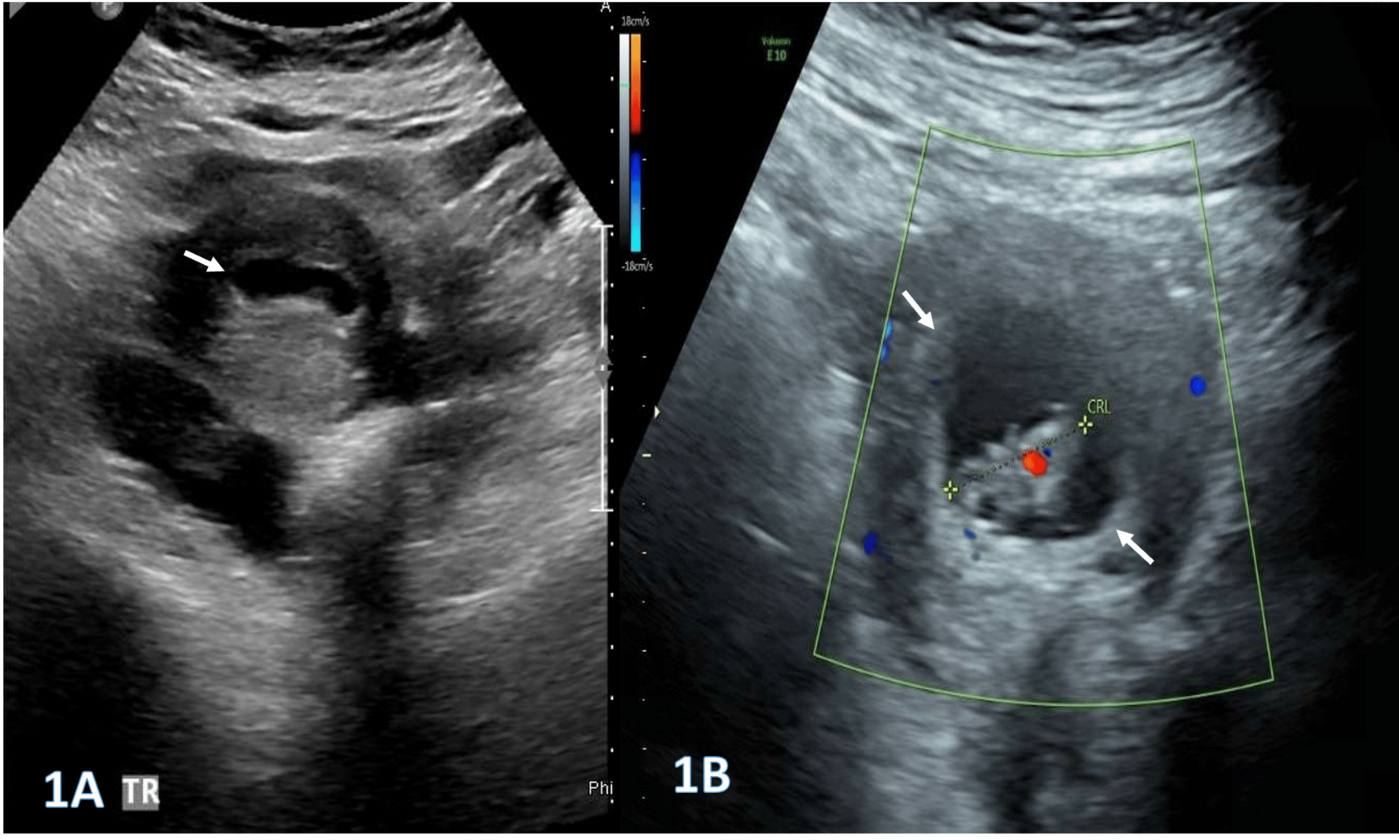
Selected pelvic ultrasound images (1A and 1B) demonstrate an early intrauterine pregnancy with an embryo with a positive heart rate and CRL corresponding to eight weeks. CRL: crown rump length

We present the case of a 41-year-old woman, eight weeks pregnant and previously healthy, with a history of three cesarean sections, who presented to the emergency department with sudden-onset severe pain in the upper left quadrant of the abdomen, radiating to the back, and associated with nausea, without other significant symptoms. On arrival, her vital signs were stable: blood pressure of 100/58 mmHg, pulse rate of 92 beats per minute, and temperature of 37°C. A gynecology follow-up performed two days earlier was reassuring, confirming a normal intrauterine pregnancy, as demonstrated in Figure 1A and Figure 1B.

**Figure 2:**
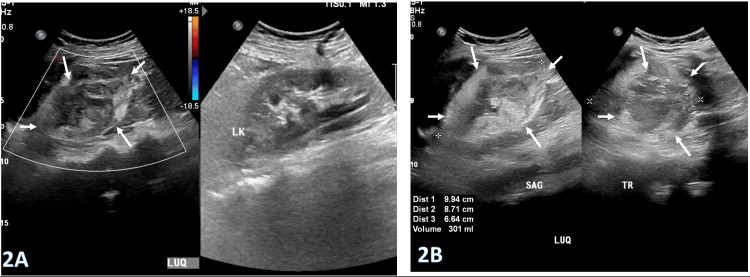
Selected ultrasound images (2A and 2B) demonstrate a large heterogeneous lesion in the left upper quadrant (LUQ) measuring 9.9 × 8.7 × 6.6 cm (arrows; approximate volume: 300 mL), with no significant internal color flow.

Her physical examination was notable for localized tenderness and guarding in the left loin, extending posteriorly toward the upper abdomen. There were no other remarkable findings. The initial differential diagnosis included renal colic, ovarian-related emergencies, splenic infarction, and heterotopic pregnancy. Ultrasonography revealed a large heterogeneous lesion in the left upper quadrant measuring 9.9 × 8.7 × 6.6 cm (estimated volume approximately 300 mL), with no significant internal color flow, as demonstrated in Figure 2A and Figure 2B.

Further diagnostic evaluation was pursued, given the patient’s acute presentation and persistent abdominal pain; therefore, an abdominal MRI was initially considered.

**Figure 3:**
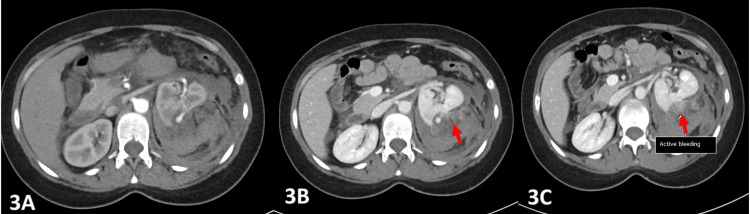
Selected contrast-enhanced axial CT images (arterial phase, 3A; venous phase, 3B; and delayed phase, 3C) demonstrate active contrast extravasation (arrow) and a well-defined saccular lesion in the upper posterior kidney. The lesion follows an arterial enhancement pattern, with avid enhancement in the arterial phase, slight increase in the venous phase, and washout on delayed imaging.

The patient remained hemodynamically stable throughout her emergency department course; however, on reassessment after about two hours, her pain persisted, and she was slightly pale and slightly tachycardic (heart rate {HR}: 105), with no drop in her blood pressure. Her abdominal examination was unchanged from previous findings, with no signs of peritonitis. A venous blood gas revealed a marked decline in hemoglobin from 10 g/dL to 7 g/dL, with an elevated lactate of 6 mmol/L. The patient underwent appropriate resuscitation with intravenous fluid (IVF) and two units of packed red blood cells. The above findings heightened concern for active intra-abdominal hemorrhage. In this emergent context, MRI was deemed unsuitable due to its prolonged acquisition time and logistical limitations. Although CT imaging carries a potential risk of fetomaternal radiation exposure, a risk-benefit assessment prioritized maternal stabilization. Accordingly, a contrast-enhanced CT scan of the abdomen was performed, demonstrating an extensive left perinephric retroperitoneal hematoma measuring approximately 350 mL. Active contrast extravasation was identified in the arterial phase, with mild progression in the venous phase and washout on delayed imaging (Figure 3A-3C).

**Figure 4:**
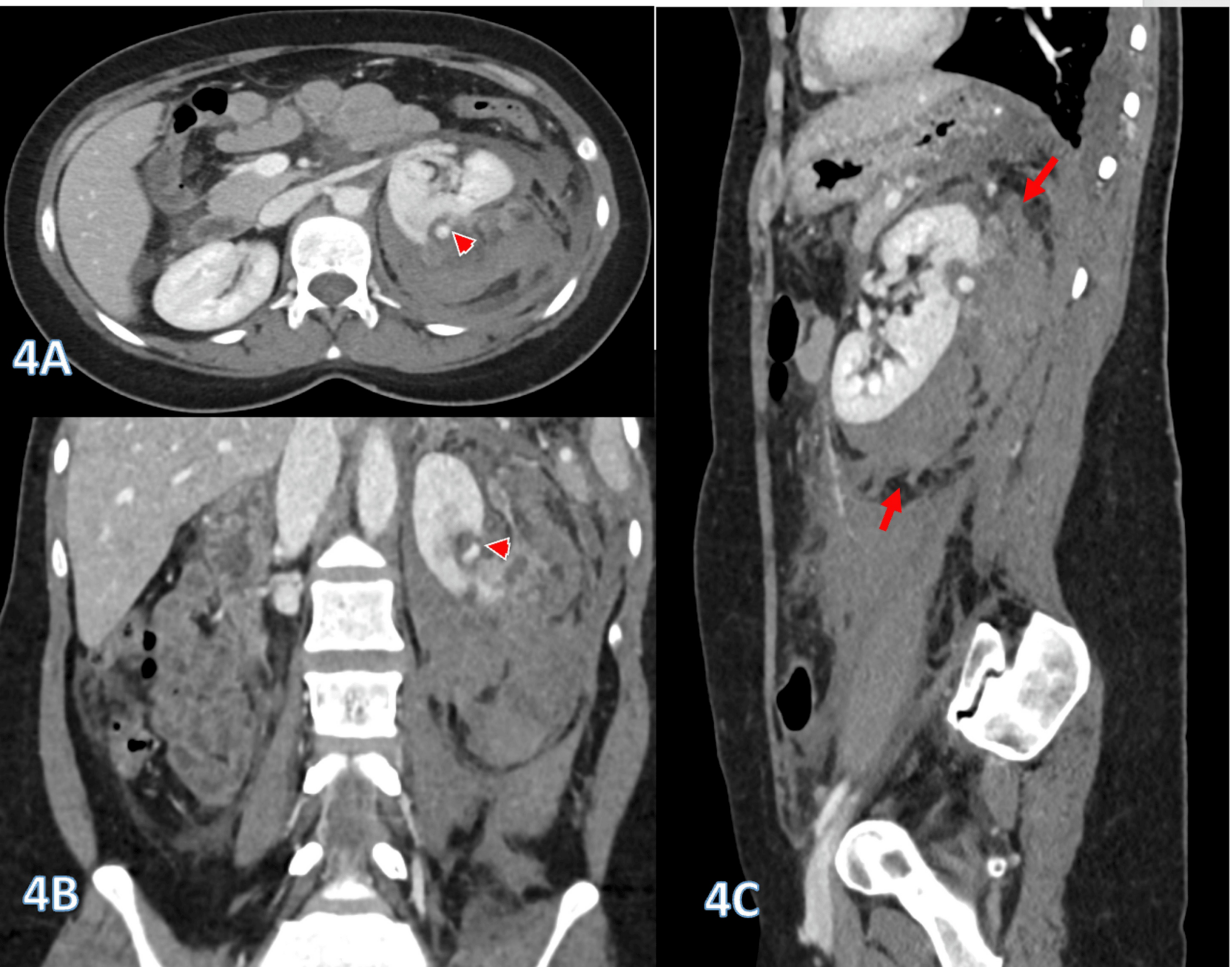
Selected contrast-enhanced CT reconstruction images (4A-4C) demonstrate an extensive perinephric retroperitoneal hematoma (arrows), with an estimated volume of approximately 350 mL and evidence of active bleeding (4B). The bunched-out renal cortex (arrowheads) suggests an underlying cortical lesion, most consistent with AML with a central saccular aneurysm. AML: angiomyolipoma

Radiological assessment suggested angiomyolipoma (AML), based on the presence of a bunched-out renal cortex indicative of an underlying cortical lesion with a central saccular aneurysm, as shown in Figure 4A-4C.

**Figure 5:**
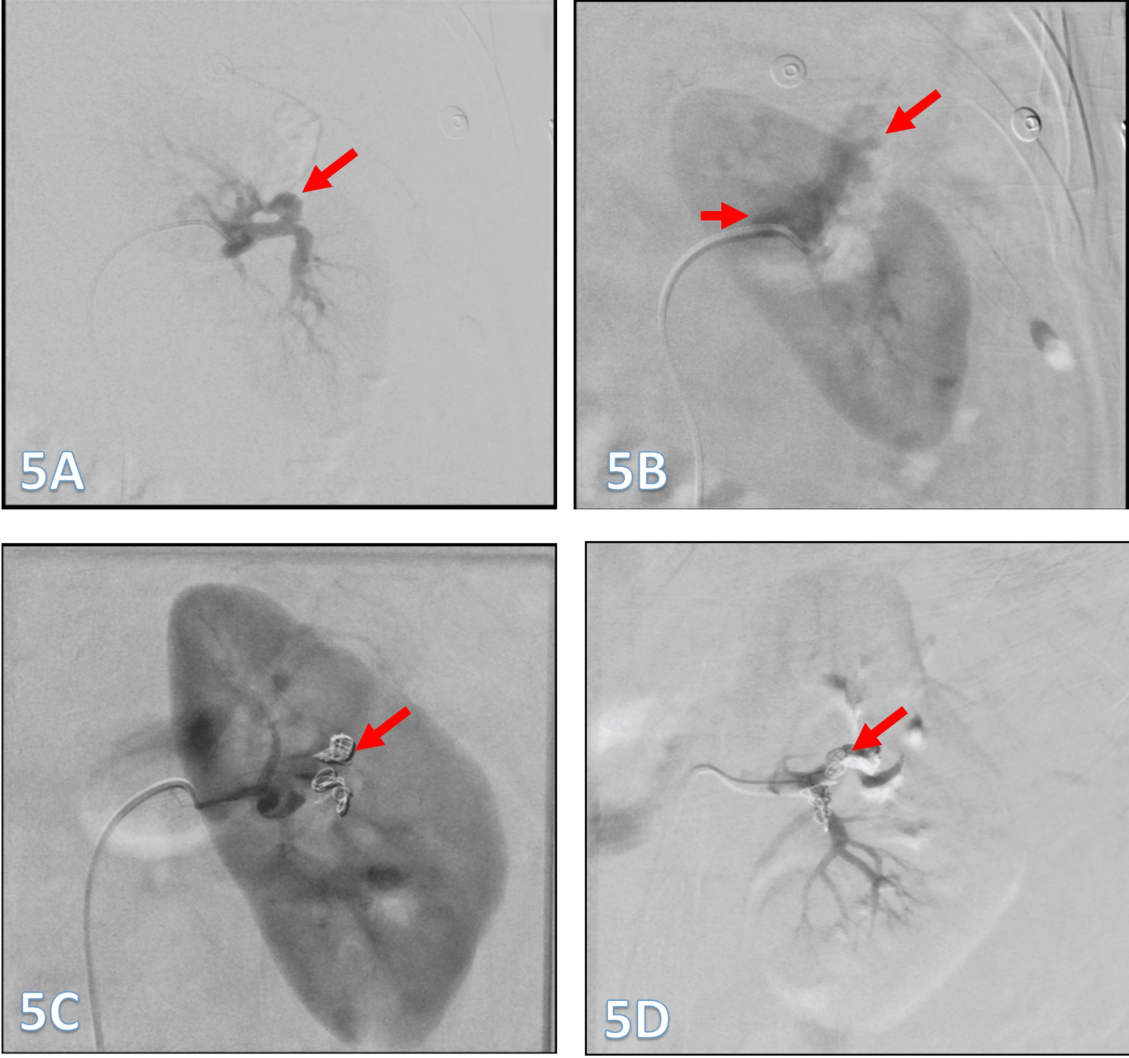
Super-selective cannulation and digital subtraction angiography (DSA) using a microcatheter demonstrate active bleeding and a pseudoaneurysm (arrow) arising from a trifurcation, with two branches contributing to the hemorrhage (5A and 5B). Super-selective embolization of the left renal artery was performed for acute bleeding due to a suspected ruptured angiomyolipoma (AML) with an intralesional pseudoaneurysm, using a combination of coils (arrows; 5B and 5C). Final DSA confirms complete occlusion with a wedge-shaped non-enhancing area corresponding to the bleeding site (arrow; 5D).

The condition was successfully treated with the endovascular embolization of the terminal branches of the left renal artery, as demonstrated by pre‑ and post‑procedure images (Figure 5A-5D). The patient was subsequently discharged home in stable condition, without any fetal or maternal complications.

## Discussion

Renal angiomyolipoma (AML) is a benign mesenchymal neoplasm composed of thick-walled blood vessels, adipose tissue, and smooth muscle cells [3,4]. Two main histological subtypes are recognized: the classic type and the epithelioid variant, the latter of which carries malignant and metastatic potential [5]. Although most renal AMLs occur as isolated lesions, approximately 20% are associated with tuberous sclerosis complex (TSC) or other phacomatoses, such as neurofibromatosis [2,6]. AMLs associated with TSC tend to be multifocal, bilateral, larger, and more prone to rupture than sporadic isolated lesions [3].

Renal AMLs are often asymptomatic and are commonly identified incidentally on ultrasonography or CT imaging [7]. However, these tumors can enlarge and develop aneurysms or rupture, resulting in life-threatening retroperitoneal hemorrhage. AMLs are known to be hormone-sensitive tumors, and pregnancy is reported to increase the risk of rupture, although the exact mechanism remains unclear [3]. Proposed contributing factors include increased abdominal pressure, hormonal influences, and structural changes within the tumor [7]. In symptomatic patients, the classic triad of flank pain, hematuria, and a palpable abdominal mass is frequently described [8]. Severe complications, such as hemorrhagic shock, may occur due to spontaneous aneurysm or tumor rupture [9]. In acute presentations, the differential diagnosis should include pyelonephritis, renal colic, ruptured renal artery aneurysm, uterine rupture, and other vascular emergencies.

The risk of AML rupture correlates with tumor size, and many studies suggest that lesions exceeding 4-5 cm carry a significantly higher likelihood of bleeding [4,10,11]. Symptomatic AMLs warrant treatment regardless of size.

The primary therapeutic goal in AML management is to prevent complications while preserving renal function. Treatment options include surgical intervention (partial or total nephrectomy) or minimally invasive selective arterial embolization [12,13]. Active intervention is indicated for progressive lesion growth on follow-up, symptomatic presentation, the suspicion of malignancy, or hemorrhagic complications such as hematuria or retroperitoneal bleeding. Although tumor size was historically a major determinant in management decisions, recent literature indicates that size alone may not reliably predict complications; instead, clinical symptoms and specific imaging characteristics should be prioritized in risk assessment [14]. At our institution, given the patient’s pregnancy and the availability of an experienced interventional radiology team, a kidney-sparing, minimally invasive strategy was preferred. Selective renal arterial embolization was therefore performed, achieving effective hemorrhage control while preserving renal function.

## Conclusions

Renal angiomyolipoma (AML) is an uncommon urological tumor with the potential for significant morbidity, particularly during pregnancy, due to its hormone-responsive nature and increased risk of rupture. The prompt recognition of high-risk features, such as rapid lesion enlargement, large aneurysms, or acute symptomatic presentation, is crucial for preventing serious maternal and fetal outcomes.

Despite concerns regarding fetal radiation exposure, contrast-enhanced CT remains the most practical and reliable diagnostic tool in emergency settings when active hemorrhage is suspected. Selective arterial embolization is a safe, effective, and kidney-preserving treatment for controlling acute bleeding. This case underscores the importance of early multidisciplinary collaboration among emergency medicine, obstetrics, radiology, and interventional teams. Clinicians should maintain a high index of suspicion for ruptured AML in pregnant patients presenting with acute flank or abdominal pain. Timely diagnosis and intervention are essential to optimizing maternal and fetal outcomes.
